# Process intensification of the baculovirus expression vector system using a perfusion process with a low multiplicity of infection at high cell concentrations

**DOI:** 10.1002/btpr.3527

**Published:** 2025-01-23

**Authors:** Jort J. Altenburg, Brenda E. Juarez‐Garza, Jelle van Keimpema, Linda van Oosten, Gorben P. Pijlman, Monique M. van Oers, René H. Wijffels, Dirk E. Martens

**Affiliations:** ^1^ Bioprocess Engineering Wageningen University & Research Wageningen The Netherlands; ^2^ Laboratory of Virology Wageningen University & Research Wageningen The Netherlands

**Keywords:** baculovirus expression vector system, insect cell culture, perfusion, process intensification

## Abstract

The emergence of new viruses and the spread of existing pathogens necessitate efficient vaccine production methods. The baculovirus expression vector system (BEVS) is an efficient and scalable system for subunit and virus‐like particle vaccine production and gene therapy vectors. However, current production processes are often limited to low cell concentrations (1–4 × 10^6^ cells/mL) in fed‐batch mode. To improve the volumetric productivity of the BEVS, a medium exchange strategy was investigated. Screening experiments were performed to test baculovirus (expressing green fluorescent protein; GFP) infection and productivity of insect cell cultures infected at high cell concentration (1–2 × 10^7^ cells/mL), showing that infection at high cell concentrations was possible with medium exchange. Next, duplicate perfusion runs with baculovirus infection were performed using a cell concentration upon infection (CCI) of 1.2 × 10^7^ cells/mL and a multiplicity of infection (MOI) of 0.01, reaching a maximum viable cell concentration of 2.8 × 10^7^ cells/mL and a maximum GFP production of 263 mg/L. The volumetric productivity of these perfusion runs was 4.8 times higher than for reference batch processes with a CCI of 3 × 10^6^ cells/mL and an MOI of 1. These results demonstrate that process intensification can be achieved for the BEVS by implementing perfusion, resulting in a higher volumetric productivity.

## 1 INTRODUCTION

Several viral outbreaks with epidemic and pandemic potential, most of which are zoonotic, have been witnessed in the last two decades.^1^ Examples include the 2002–2003 outbreak of SARS, the 2009 outbreak of H1N1 influenza (swine flu), the 2013–2016 outbreak of Ebola, and the 2019–2020 outbreak of COVID‐19 caused by SARS‐CoV‐2. It is expected that the emergence of new infectious diseases will become more common due to social, environmental, and economic factors.^2^ Vaccines are the most effective medical intervention to combat global viral outbreaks.^3^ To effectively contain a viral outbreak, the timely availability of vaccine material in sufficient amounts is crucial. Consequently, the ability to quickly develop and efficiently scale‐up vaccine production processes is critical. The baculovirus expression vector system (BEVS) allows the expression of foreign genes when infecting insect cells with a recombinant baculovirus. In this system, the baculovirus polyhedrin gene, which is expressed at a high level and is not essential for virus replication, is replaced by a gene of choice.^4^ The infection process and protein production using the BEVS is significantly influenced by the multiplicity of infection (MOI) and the cell concentration at infection (CCI). The MOI is the number of infectious baculoviruses per cell added to a culture.^5^ In industrial processes a MOI larger than 5 is generally considered a high MOI and a MOI lower than one a low MOI. A high MOI ensures the immediate infection of all insect cells upon virus addition. Infecting at a too‐high CCI leads to reduced protein production due to the cell density effect, while infection at a too‐low CCI results in limited protein production due to the small concentration of infected cells. With a high MOI approach, infection at the optimal CCI is easily attainable, but a large amount of virus particles is needed.^6^ The use of a low MOI strategy requires multiple infection cycles in the process and enables the use of smaller virus stocks. If with a low MOI the CCI is chosen too high, cells will grow to too high cell concentrations leading to decreased protein production due to the cell density effect. When the CCI is chosen too low, full infection will occur at low cell concentrations also leading to reduced protein production. Although these general principles are well known, optimal values for CCI and MOI vary with the virus and cell line used. Thus, knowledge on the interplay between MOI and CCI, including infection and growth kinetics, for the specific cell line and virus used is essential for designing efficient infection strategies.^5^, ^6^

Batch culture is commonly used to produce recombinant proteins with baculovirus‐infected insect cells although more recently perfusion culture has been applied for recombinant protein production using stable insect cell lines.^7^ Batch processes have a low volumetric productivity because of a low optimal CCI and relatively long downtime of the equipment due to the short duration of a batch.^8^ Perfusion processes are an attractive alternative to produce recombinant proteins, because high cell concentrations and consequently high volumetric productivities can be reached.^9^

To achieve a high volumetric productivity with the BEVS in a perfusion bioreactor, it is beneficial to acquire high cell concentrations during the production run, provided that the specific productivity remains constant. However, it has been observed that there is a reduction in specific productivity when infecting insect cells at cell concentrations above 5 × 10^6^ cells/mL.^10^, ^11^ This phenomenon is often referred to as the cell density effect. Potential explanations for this cell density effect are cells being in an unfavorable growth phase at the moment of infection, inhibition by accumulating waste metabolites, cell‐to‐cell contact inhibition, disruption in lipid biosynthesis, and the depletion of essential nutrients.^10^, ^12^, ^13^ Although the exact mechanisms behind the cell density effect are still unknown, it has been prevented by a continuous supply of nutrients and/or the removal of inhibitory products.^12^, ^14^, ^15^, ^16^ Carinhas et al.^5^ showed that medium exchange could overcome the cell density effect at high MOI but not at low MOI for the Sf9 cell line in SF900II serum‐free medium. This previous research on the use of medium exchange or perfusion with the BEVS was performed with various cell lines and media types (including serum‐containing medium) at relatively low cell concentrations and employed high MOIs for baculovirus infection. Until now, the maximum reported CCIs for Sf9 cells in batch mode are generally in the range of 1–3.5·10^6^ cells/mL at MOIs >1.^13^

This study aimed to investigate whether it is possible to achieve high cell concentration protein production for the BEVS by culturing cells in perfusion mode in bioreactors equipped with an acoustic filter using a high CCI and low MOI for infection. First, small‐scale screening experiments with ExpiSf9 insect cells in ExpiSf chemically defined medium were performed in shake flasks and spin tubes to determine the feasibility of baculovirus infection at high cell concentrations and to see whether cell‐specific productivity is maintained. Hereafter, the process was scaled‐up to a stirred tank bioreactor with a working volume of 300 mL, and a low MOI perfusion strategy for the BEVS was applied using an acoustic cell retention device. Viable cell concentrations of 2.5–2.8 × 10^7^ cells/mL were achieved in two acoustic perfusion bioreactor runs that were highly reproducible. We showed that yield per cell is maintained at these high cell concentrations and bioreactor volumetric productivity was over 4.8‐fold higher compared to reference batch processes.

## 2 MATERIALS AND METHODS

### 2.1 Cell lines, media, and virus stocks

The ExpiSf9 cell line (ThermoFisher) was used for all the experiments and for the end‐point dilution assays (EPDA). This cell line is a derivative of the *Spodoptera frugiperda* (Sf9) cell line that was adapted to grow on the chemically defined ExpiSF CD medium by Gibco (ThermoFisher, 2018). An *Autographa californica* multiple nucleopolyhedrovirus (AcMNPV) vector encoding two fluorescent markers was constructed. This virus (Ac‐2FL) contains the eGFP open reading frame (ORF) behind the *Orchyia pseudotsugata* MNPV immediate‐early 2 (OpIE‐2) promoter as a marker for early infection^17^ and a mCherry ORF behind the polyhedrin promoter as a marker for very late gene expression, and hence, protein productivity. For the experiments involving protein quantification, an AcMNPV vector containing the GFP ORF behind the polyhedrin promoter was used (AcGFP). The recombinant baculoviruses were generated using the bac‐to‐bac expression system (Thermo Fisher) and had a titer of 1–2 × 10^7^ TCID50/mL. In short, *Escherichia coli* cells containing the AcMNPV bacmid and the transposition helper plasmid were transformed with pDest8‐derived plasmids to introduce the marker genes into the baculovirus genome. Insect cells were then transfected with the recombinant bacmids using Expres2TR transfection reagent (Expres2ion Biotechnologies) to generate infectious baculovirus particles.

### 2.2 Screening and bioreactor cultures

Screening experiments were performed by cultivating cells in 125 mL vent‐capped unbaffled PETG shake flasks (Nalgene) with a working volume of 25 mL or by cultivating in 50 mL spin tubes (Sarstedt) with a working volume of 5 mL. The shake flasks were incubated in a Climo‐Shaker ISF1‐XC (Kuhner) at 27°C with a shake velocity of 95 rpm and an orbital diameter of 25 mm. The spin tubes were mixed at a shake velocity of 240 rpm and an orbital diameter of 20 mm on a Rotamax 120 (Heidolph) shaker placed within a Multitron (Infors HT) incubator at 27°C without CO_2_. Medium exchange was performed by centrifuging the cell suspension at 300 g for 5 min. Medium exchange was started when a cell concentration of 5–6 × 10^6^ cells/mL was reached, and 30% of the medium was replaced once a day. After reaching a viable cell concentration (VCD) of 1.1–1.2 × 10^7^ cells/mL, 60% of the medium was replaced once a day (corresponding to an approximate continuous perfusion rate of 0.5 and 1.0 reactor volumes (RV)/day). For infection experiments, the medium replacement was done just before the infection.

Bioreactor experiments were performed in 500 mL miniBio stirred tank bioreactors (Getinge) using a my‐Control bioreactor control system (Getinge). The reactor working volume of 300 mL was inoculated at a starting concentration of 1.0 × 10^6^ viable cells/mL. The temperature was controlled at 27°C using a Peltier heating/cooling system. The pH was not controlled but maintained between 5.6 and 6.2 by the buffering capacity of the medium. Dissolved oxygen was controlled at 30% of air saturation by supplying pure oxygen through an open hole sparger with a 2 mm internal diameter (Getinge). A single 25 mm marine impeller (Getinge) operated at 300 rpm provided agitation. Perfusion was initiated when the viable cell concentration reached 4.0–7.0 × 10^6^ viable cells/mL. The targeted cell‐specific perfusion rate (CSPR) was 50 pL/cell/day. Cell retention was achieved by acoustic filtration using a 1 L BioSep and APS 990 controller (Getinge). The controller was set at run/stop cycle times of 10 min/3 s and a power level of 2 W. The perfusion rate was set using a Watson‐Marlow 205 U (Watson‐Marlow) peristaltic pump connected to the APS990 controller. In this way, the pump was paused during the stop cycle time. The recirculation flow was operated at 1.5 mL/min. The feed pump was controlled by the my‐Control system and activated based on a level sensor to ensure a constant reactor volume. The off‐gas flow was connected to a mass spectrometer (Thermo Fisher).

### 2.3 Analytical methods

Cells were counted by trypan blue exclusion using a TC20 Automated Cell Counter (Bio‐Rad) or by manual counting using DHC‐F01 cell counting chambers (INCYTO). Infected cells were visualized by expression of GFP or mCherry detected by an IX71 fluorescent microscope (Olympus) or by a C6 Plus Flow Cytometer (BD Accuri). Bioreactor off‐gas composition was measured with a mass spectrometer (Thermo Fisher). GFP concentrations were measured by comparing fluorescence to a GFP standard solution (Thermo Fisher). Fluorescence was quantified using an FLx800 micro‐plate reader (BioTek) with a 485/528 excitation/emission filter. To determine intracellular GFP, 250 μL lysis buffer (500 mM NaCl, 50 mM Tris(hydroxymethyl)methylamine (TRIS), 0.1% Triton X‐100, pH 8) supplemented with 1 mM Phenylmethylsulfonyl fluoride (PMSF) was added to 100 μL of cell‐containing sample fluid. This mixture was incubated on ice for 10 min and subsequently centrifuged at 13000 g for 10 min. The supernatant was stored at −20°C until further use. All baculovirus stocks were amplified in ExpiSf9 cells. The infectious viral titer of the produced baculovirus stocks and cell culture samples were determined by calculating the median 50% tissue culture infectious dose (TCID_50_) per mL via end‐point dilution assay and had values of 1–2 × 10^7^ TCID50/ml.

### 2.4 Calculations

#### 2.4.1 Separation efficiency

The separation efficiency (%) of the acoustic cell separator was calculated considering the total cell concentration in the reactor (TCC_R_, cells/mL) and the harvest flow (TCC_H_, cells/mL).

(1) $$\mathrm{SE}=\left(1-\frac{{\mathrm{TCC}}_{H}}{{\mathrm{TCC}}_{R}}\right)\times 100.$$

#### 2.4.2 Cell specific perfusion rate

The CSPR (m^3^/cell^/^day), is defined as the amount of medium supplied to a single viable cell per day and is given by:

(2) $$\text{CSPR}=\frac{P}{\mathrm{VCC}},$$

where *P* is the perfusion rate (day^−1^), which is the total medium flow through the cell retention device (m^3^/day) divided by the reactor working volume (m^3^) and VCC is the viable cell concentration (cells/m^3^).

#### 2.4.3 Yield of product per cell

The yield of product per cell (*Y*_P/C_) (μg/cell) is defined as:

(3) $$Y_{P/C}=\frac{m_{P}}{{\mathrm{VCC}}_{\mathrm{MAX}}},$$

where m_P_ is the total amount of product produced since the start of the process (μg) and VCC_max_ is the maximum number of viable cells reached in the reactor (cells).

#### 2.4.4 Yield of product per medium

The yield of product per medium (*Y*_P/M_) (μg/mL) was calculated according to:

(4) $$Y_{P/M}=\frac{m_{P}}{V_{m}},$$

where V_m_ is the total amount of medium used (mL).

#### 2.4.5 Volumetric productivity

The volumetric productivity (mg/L/day) is calculated according to:

(5) $$\mathrm{VP}=\frac{m_{P}}{t∙V_{R}},$$

where *t* is the process duration (days) and *V*_R_ is the working volume of the reactor (L).

#### 2.4.6 Specific productivity

The specific productivity (mg/cell/day) is calculated according to:

$$q_{p}=\frac{m_{P}\left(t\right)-m_{P}\left(t-1\right)}{{\mathrm{VCC}}_{\mathrm{av}}∙∆t},$$

where VCC_av_ is the average cell viable cell concentration over the period (cells/ml) and Δt is the time period (days).

## 3 RESULTS AND DISCUSSION

### 3.1 Baculovirus infection at high Sf9 cell concentrations

**FIGURE 1:**
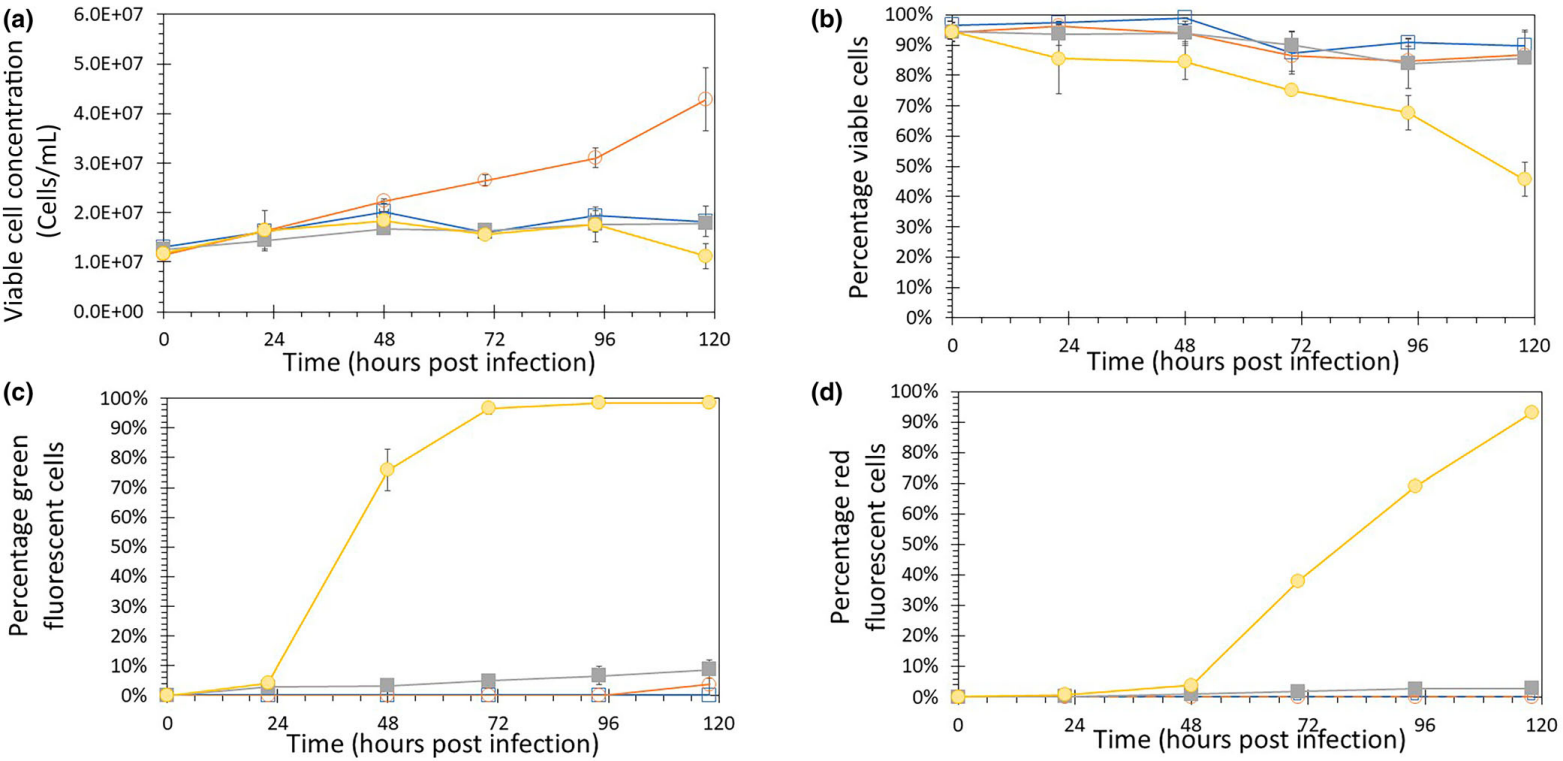
Shake flask Sf9 cultures infected with baculovirus at high cell concentrations. The control Sf9 culture was grown without medium exchange or baculovirus infection (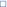). A second control was grown with medium exchange but without baculovirus infection (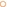). The infected shake flasks were infected with the Ac‐2FL baculovirus, expressing eGFP from the immediate early OpIE2 promoter and mCherry from the very late polyhedrin promoter. Cells were infected at 1.2 × 10^7^ viable cells/mL at an MOI of 0.01 baculovirus/cell. Infected shake flasks either had; no medium exchange (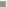) or daily medium exchange (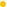). (a) Viable cell concentrations. (b) Percentage of viable cells. (c) Percentage of cells containing early expressed eGFP. (d) Percentage of cells containing late expressed mCherry. The data shown are averages of duplicate runs. and error bars represent the distance to the minimum and maximum value Data are shown starting from 1.2 × 107 viable cells/mL, which was the moment the cells were infected.

The loss in productivity occurring after infection of Sf9 cells above a certain cell concentration has been identified as a bottleneck for the BEVS.^5^, ^11^, ^18^ To test whether this bottleneck is due to nutrient limitation or inhibition of produced metabolites we applied an exchange strategy with ExpiSF CD medium to shake flask cultures. First, we determined in shake flasks whether non‐infected cells can grow to higher cell concentrations. For this two shake flasks cultivations without medium exchange and two shake flasks cultivations with medium exchange were performed. The maximum viable cell concentration of these non‐infected cultures with medium exchange reached 4.2 × 10^7^ viable cells/mL, which is twice the maximum viable cell concentration reported in literature for this cell line in batch culture^19^ and more than twice the viable cell concentration of 1.8 × 10^7^ cells/mL reached by the batch control culture without medium exchange (Figure 1a). This indicated that the maximum viable cell concentration can be increased by supplying additional nutrients required for the growth and maintenance of these cells and/or by removal of growth‐inhibiting metabolites with the spent medium. For both cultures, the viability stayed above 80% during the entire run (Figure 1b).

Next, we determined whether complete infection can be achieved when cells are infected at high concentrations when applying medium exchange. For all cultures, a medium exchange was done just before the infection, after which the next exchange was done the next day. For the infected shake flasks, a baculovirus encoding two fluorescent markers (Ac‐2FL) was used, containing a gene for GFP located behind the immediate early OpIE2 promoter and a mCherry gene located behind the very late polyhedrin promoter. Infection was evaluated by measuring the percentages of GFP and mCherry fluorescent cells.

In batch mode, the optimal CCI for these cells was 5–7 × 10^6^ cells/mL when infected with an MOI of 5.^19^ Here, the cell concentration upon infection was 1.2 × 10^7^ cells/mL, twice the average recommended cell concentration for infection. The cells were infected with AcBac‐2FL at an MOI of 0.01, allowing for the continued growth of non‐infected cells after infection. Four shake flasks were infected in total, two without medium exchange and two with medium exchange.

The viable cell concentration of the infected shake flasks without medium exchange was similar to the uninfected batch control without medium exchange (Figure 1a). This was expected because infecting at low MOI requires more time to infect all cells. Cells continued to grow eventually reaching the maximum cell concentration for a batch culture of 1.8 × 10^7^ cells/mL before all cells were infected. Viability was maintained above 80%, which is comparable to that of the non‐infected control (Figure 1b). The infected culture without medium exchange only reached very low levels of green and red fluorescent cells, indicating that the infection was halted (Figure 1c,d). These experiments show that for a batch process using a low MOI (0.01) a CCI of 1.2 × 10^7^ cells/mL is too high, resulting in cells growing to the maximum cell concentration and an infection percentage below 10%.

The infected shake flasks with medium exchange had comparable growth to the control with medium exchange until 20 h (Figure 1a). This was expected since the low MOI of 0.01 requires multiple infection cycles before all cells are infected, allowing for continued cell growth. The maximum viable cell concentration of 1.6 × 10^7^ viable cells/mL was reached at 48 h post‐infection (hpi). This is considerably lower than the maximum concentration of 4.0 × 10^7^ in the non‐infected control with medium exchange indicating a stop of cell growth due to baculovirus infection. In contrast to the other conditions, the infected shake flasks with medium exchange showed a drop in viable cell concentration between 100 and 120 hpi and had a significant reduction in viability, reaching around 45% after 120 hpi. A reduction in viable cell concentration and viability is expected when insect cells undergo baculovirus infection. Fluorescence measurements indicated an efficient infection for the shake flasks with medium exchange, with infection percentages of 100% based on the early expression of eGFP (Figure 1c) and 93% based on the late expression of mCherry (Figure 1d). This demonstrated that medium exchange enabled baculovirus infection of all cells in the culture at high cell concentrations while using a low MOI. For High MOI infections (MOI 5 or higher) 100% green fluorescence is typically reached before 24 h.

A comparison between eGFP and mCherry expression showed that eGFP started to be detected at 22 hpi, while mCherry was seen after 46 h. The difference in time of expression is expected because the eGFP gene is behind the immediate early baculovirus promoter OpIE‐2 and the mCherry gene is behind the very late polyhedrin promoter. In summary, these experiments showed that medium exchange allowed for complete baculovirus infection at high cell concentrations when infecting with a low MOI.

### 3.2 Productivity at high cell concentrations with medium exchange

**FIGURE 2:**
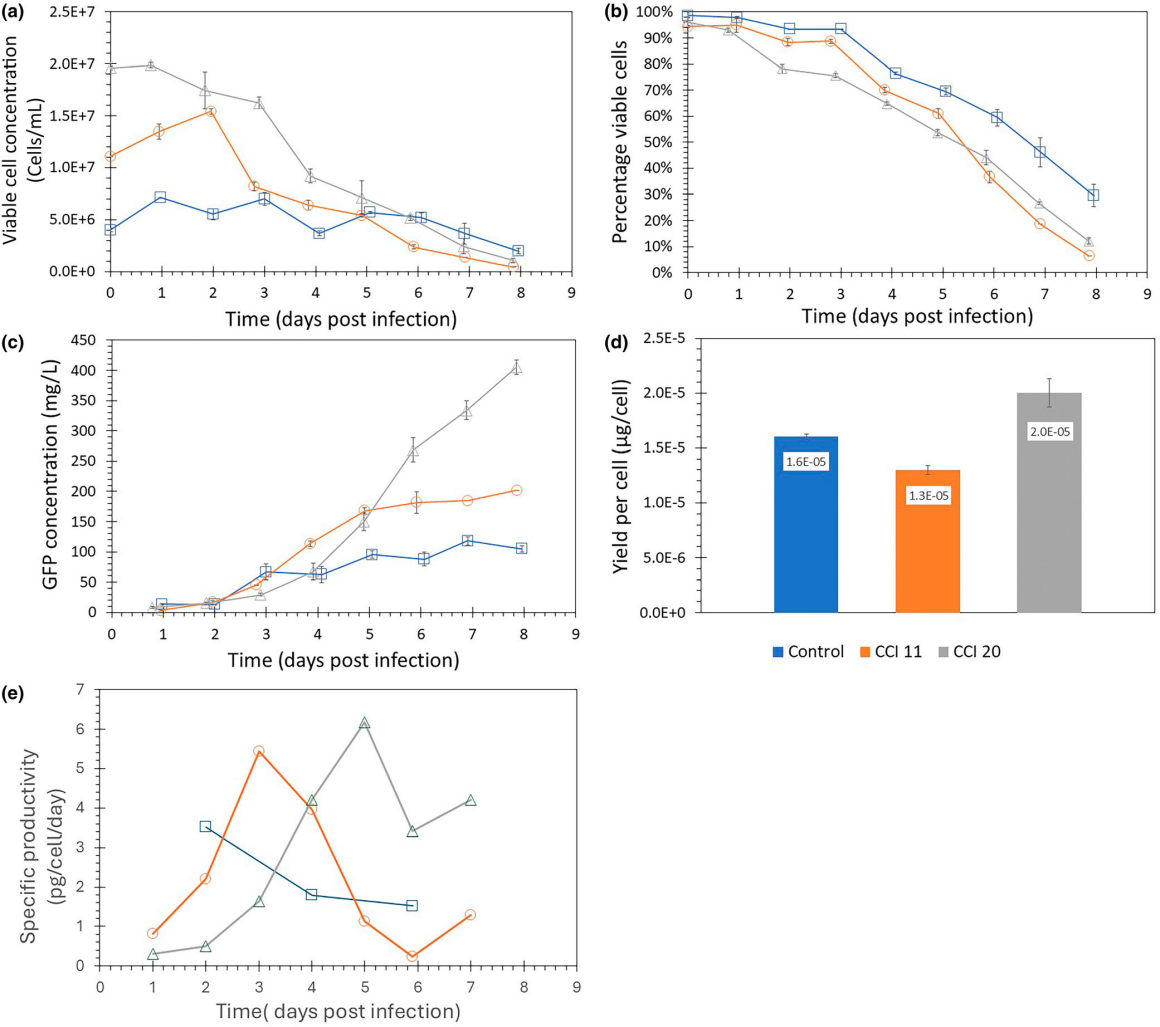
Productivity of Sf9 cultures in spin tubes at different cell concentration upon infections (CCIs). Spinner tubes were infected at an multiplicity of infection (MOI) of 0.1 at CCIs of 4 × 10^6^ viable cells/mL (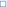), 1.1 × 10^7^ viable cells/mL (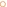), and 2.0 × 10^7^ viable cells/mL (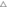). Medium exchange was performed once per day for CCIs 1.1 × 10^7^ viable cells/mL (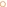), and 2.0 × 10^7^ viable cells/mL (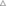). The cells were infected with an AcGFP baculovirus. Data shown are averages of duplicate runs and error bars represent the distance to the minimum and maximum value. (a) Viable cell concentrations. (b) Viability. (c) Green fluorescent protein (GFP) concentration. (d) Yield of product per cell (*Y*_p/c_) (e) Specific productivity of infected cultures with an MOI of 0.1 in spin tubes.

Next, we studied whether the product yield per cell could be maintained when infecting cells at higher concentrations while applying medium exchange. High cell‐ concentration baculovirus infections were performed using spin tubes. The cells were infected at an MOI of 0.1 (Higher MOIs would require too much virus stock volume to be added) with AcGFP, and GFP production was quantified by fluorescence intensity measurements. The experiment included a control without medium exchange that was infected at a low cell concentration of 4 × 10^6^ cells/mL, which is around or just below the optimal CCI for these conditions, and two experimental cultures with 60% medium exchange per day that were infected at higher concentrations of respectively 1.1 × 10^7^ and 2.0 × 10^7^ cells/mL. The infection was done directly after the medium exchange after which the next medium exchange was done the following day. Since cells were infected with an MOI of 0.1, the viable cell concentration continued to increase after infection (Figure 2a). For the control flasks infected at 4 × 10^6^ cells/mL the maximum cell concentration reached was around 7.1 × 10^6^ cells/mL, a 79% increase compared to the initial cell concentration at the moment of infection. Similarly, the cell concentration increased for the cultures infected at 1.1 × 10^7^ and 2.0 × 10^7^ cells/mL. However, the increase in cell concentration after infection was reduced to 39% and 1.5%, respectively. The viability after infection dropped more drastically for the cultures infected at 1.1 × 10^7^ and 2.0 × 10^7^ cells/mL compared to the control culture that was infected at 4 × 10^6^ cells/mL (Figure 2b). At 8 days post‐infection, the viability of the control culture was around 30%, while the viabilities for the cultures infected at 1.1 × 10^7^ and 2.0 × 10^7^ cells/mL were 6% and 12%, respectively. Both the reduced growth and faster drop in viability could indicate nutrient or oxygen limitation. While at 4 × 10^6^ cells/mL, the cells were still growing exponentially, at 1.1 × 10^7^ and 2.0 × 10^7^ cells/mL the cells no longer grew exponentially and the specific growth decreased with increasing cell concentration. Exposing the cells to a sufficiently high medium exchange rate is important to maintain cell growth.^20^ To compare the productivity the total concentration (intracellular and extracellular) of GFP was measured (Figure 2c). GFP was first detected on day 1 post‐infection which is expected when using an MOI of 0.1 and the GFP gene expressed from the polyhedrin promoter. The culture infected at 1.1 × 10^7^ cells/mL showed a similar GFP production pattern compared to the control culture and maximum GFP concentration leveled off at around 200 mg/L compared to 100 mg/L for the control. The culture infected at 2.0 × 10^7^ cells/mL had lower GFP production in the beginning compared to the other two cultures. Moreover, GFP concentrations did not reach a plateau value and were still increasing until 8 days post‐infection. Possibly, at these high cell concentrations, insufficient medium exchange rates slowed down GFP production. The total amount of GFP produced at day 8 increased with the increase of the cell concentration upon infection (Figure 2c). The *Y*_P/C_ reached by the culture infected at 1.1 × 10^7^ cells/mL was slightly lower than the control infected at 4 × 10^6^ cells/mL, while the *Y*_P/C_ reached by the culture infected at 2.0 × 10^7^ cells/mL was slightly higher than the control (Figure 2d). Viable cell concentration for the culture infected at 2.0 × 10^7^ cells/mL was maintained but did not increase after infection. This could explain the higher *Y*_P/C_ values for this CCI as fewer nutrients were used for cell division. High MOI infections could reduce the uncertainty related to additional infection cycles and give a clearer picture of the maximum CCI. However, this is not always possible at these high cell concentrations due to the large volume of virus stock required to infect at high MOI and high CCI calculated *Y*_P/C_ values were in the same order of magnitude for all CCIs, which indicates that the yield of recombinant product per cell can be maintained when infecting at a high cell concentration using medium exchange for this cell line and medium. The production seems to shift to later in the infection as the cell concentration increases as the maximum specific productivity occurs at a later moment (Figure 2e). In addition, low MOI infections seem possible. This provides a good basis for the application of medium exchange and/or perfusion strategies in combination with low MOI infection on a larger scale to intensify production.

### 3.3 Cell growth characterization in a perfusion bioreactor

**FIGURE 3:**
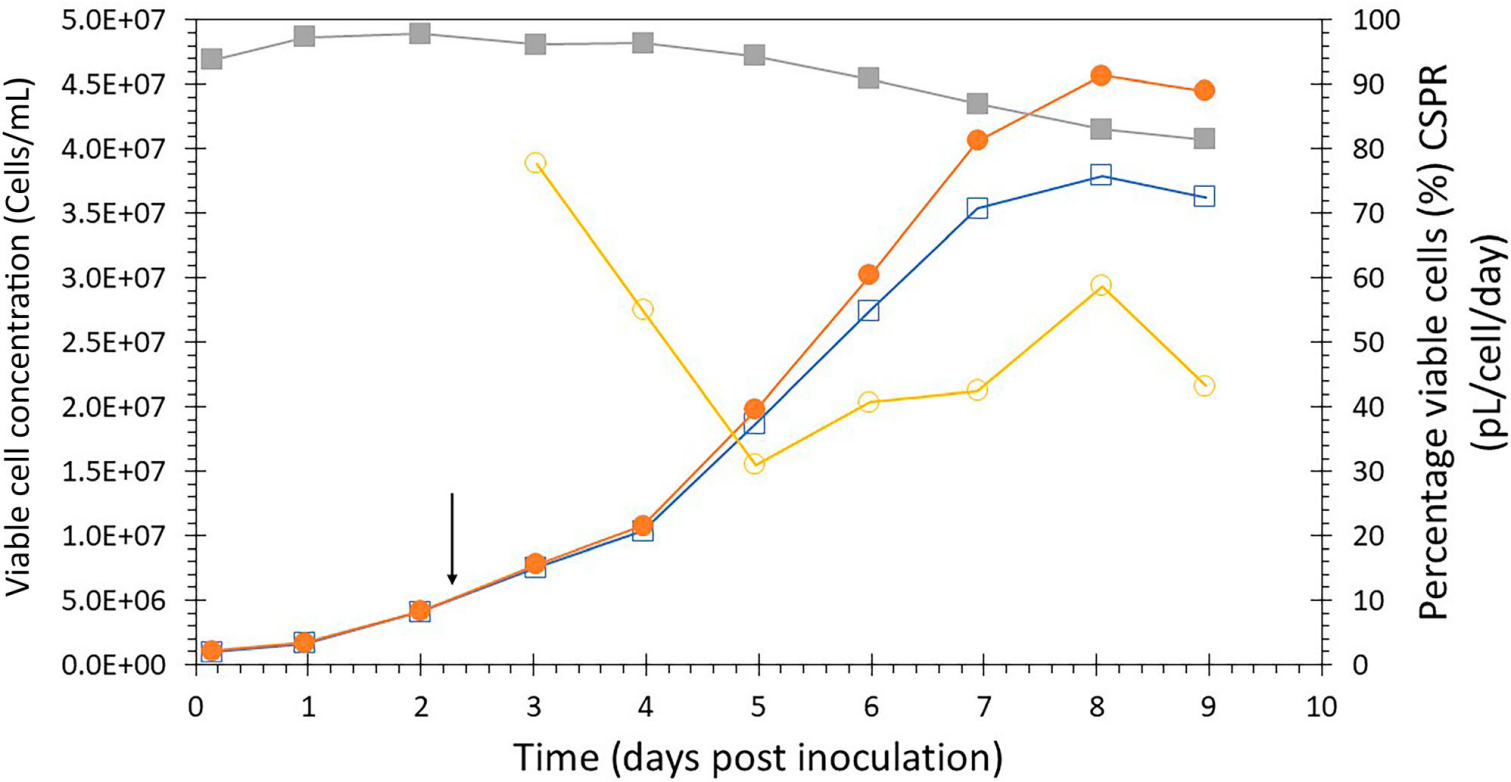
Sf9 cell growth characterization in a bioreactor run in perfusion mode. Viable (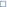) and total (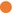) cell concentrations, percentage of viable cells (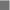), and cell‐specific perfusion rate (CSPR) (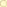) during a bioreactor run without baculovirus infection. The start of perfusion mode at day 2 is indicated by the arrow. The culture was started in batch mode with an inoculation concentration of 1.0 × 10^6^ viable cells/mL. Perfusion was started at 0.5 RV/day. The perfusion rate was further increased to 1 RV/day from day 5 onwards. On day 7, a bleed rate of 0.2 RV/day was applied to maintain viability.^21^

To scale up and test a medium exchange strategy with low MOI infection, an acoustic cell retention system was used to enable perfusion cultivation in benchtop stirred‐tank bioreactors. First, Sf9 insect cell growth was characterized in this perfusion system without baculovirus infection. The culture was started in batch mode with an inoculation concentration of 1.0 × 10^6^ viable cells/mL. Perfusion was started at 2 days post‐inoculation at 0.5 RV/day. The perfusion rate was further increased to 1 RV/day from day 5 onwards.^21^ The separation efficiency of the Mini BioSep was >95% during most of the run (Figure 3). The cell‐specific perfusion rate (CSPR) indicates the amount of medium supplied per cell per day via perfusion and must be above a certain value to prevent nutrient limitation and the associated reduction in cell growth and increase in cell death. The CSPR during the run was between 31 and 77 pL/cell/day, with an average of 50 pL/cell/day (Figure 3). While the minimum CSPR for these cells is not known, a value of 50 pL/cell/day is often used for CHO cell cultivations^20^ although lower CSPR values have been reported for stable insect cell lines.^22^ Starting on day 4, viability dropped from 95% to 80%. During this period, the CSPR dropped to 30–40 pL/cell/day and growth rates shifted from exponential to linear. It is possible that the CSPR was too low to maintain exponential growth. This could also have caused an increase in cell death and decrease in viability. Therefore, to maintain viability, a bleed rate of 0.2 RV/day was applied from day 7 onwards. This results in less increase of the viable cell concentration and thus, with a fixed perfusion rate, prevents a further decrease of the CSPR and the associated increase in cell death. In the end, a maximum viable cell concentration of 3.8 × 10^7^ cells/mL was reached at a viability of 80%. Lastly, the gas flow needed to supply sufficient oxygen increased to very high values (0.5–1 vvm, data not shown), and the shear associated with these high gas flow rates could have affected cell growth and viability. An open hole sparger generating larger 2–3 mm bubbles was used here. Switching to a porous sparger generating smaller bubbles might reduce the need for excessive gas flow rates needed for oxygenation of the cell culture and reduce shear. Overall, these results indicated a medium exchange strategy could be scaled‐up to bioreactors using an acoustic perfusion system to achieve ExpiSf9 cell concentrations of up to 3.8 × 10^7^ viable cells/mL.

### 3.4 BEVS performance in perfusion bioreactors

#### 3.4.1 Cell growth and viability

**FIGURE 4:**
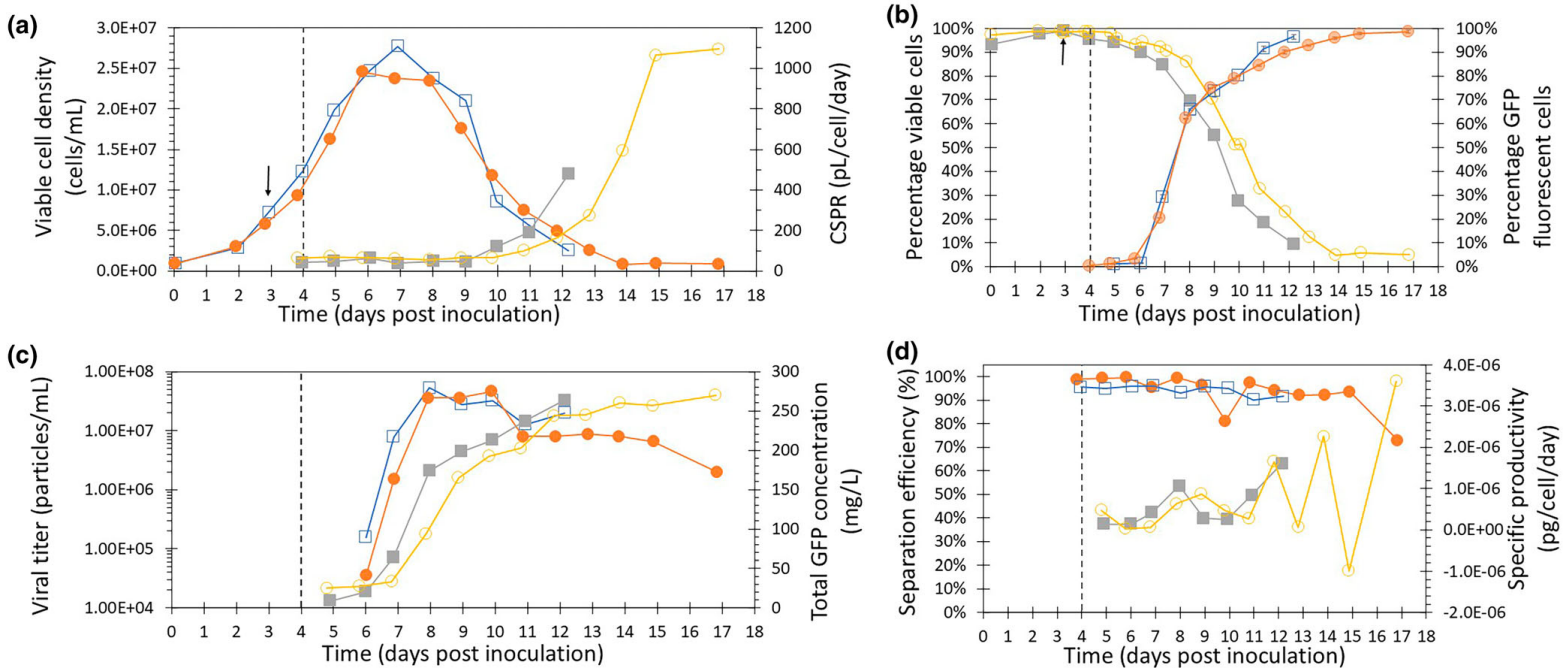
Infection of sf9 culture in bioreactors at high cell concentration and low multiplicity of infection (MOI) during perfusion cultivation mode. Two perfusion runs were performed, the reactors were started in batch mode and perfusion was started on day 3 indicated by the arrows. Insect cells were infected with baculovirus on day 4 indicated by the dashed lines. Run 1 lasted until day 12, run 2 was extended until day 17. (a) Viable cell concentrations for run 1 (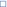), and run 2 (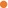), and CSPR for run 1 (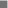), and run 2 (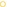). (b) Percentage of viable cells for run 1 (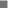), run 2 (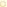), and percentage of green fluorescent protein (GFP) fluorescent cells for run 1 (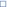), and run 2 (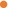). (c) Viral titer for run 1 (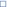), and run 2 (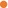) and total GFP concentration for run 1 (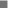), and run 2 (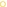). (d) Acoustic separator efficiency and specific productivity for run 1 (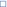), and run 2 (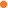).

We next investigated whether efficient baculovirus infection was possible at high cell concentration in perfusion bioreactors. Based on the cell growth characterization in the perfusion bioreactor without infection (Figure 3), two additional perfusion runs were performed with infection in 500 mL stirred tank reactors using acoustic cell retention. Both perfusion runs were inoculated with a seeding concentration of 1.0 × 10^6^ viable cells/mL. In the first days cell growth was slightly less as compared to the uninfected perfusion cultures. Therefore perfusion was started 1 day later at 3 days post‐inoculation with a target CSPR of 50 pL/cell/day, since lower CSPRs appeared to slow down cell growth. The perfusion cultures were infected at 4 days post‐inoculation. Previous experiments showed that infection was still possible at 2 × 10^7^ viable cells/mL. A doubling in cell concentrations was observed when using an MOI of 0.01. Therefore, the targeted CCI was 1 × 10^7^ viable cells/mL. Concentrations at infection were 1–1.2 × 10^7^ viable cells/mL for run 1 and 2, respectively, and both bioreactors were infected with AcGFP at low MOI (0.01). At 6–7 days post‐inoculation (corresponding to 2–3 days post‐infection) cell growth stopped, indicating complete infection, and the maximum viable cell concentration was reached (Figure 4a). Maximum viable cell concentrations were very comparable for both runs (2.5–2.8 × 10^7^ viable cells/mL). Both cell cultures doubled more than once in cell concentration after infecting with low MOI. The major part of the BV is released between 16 and 32 hpi. Assuming a replication cycle of 24 h the continued growth for about 2 days indicates that about two infection cycles were needed to reach complete infection. Maximum viable cell concentrations were substantially higher than those reached in batch mode (Table 1). Both runs were terminated at below 10% viability (Figure 4b). The viability of both runs started decreasing more rapidly from day 3 post‐infection. The viability of run 1 dropped slightly earlier than that of run 2, which may indicate a faster infection rate in run 1. Also, the lower CSPR in run 1 (Figure 4a) may have contributed to the earlier decrease in viability by causing nutrient limitation and an increase in death rate. The goal was to maintain a CSPR above 50 pL/cell/day. For run 1, the CSPR decreased below the aimed minimal value several times, with a minimum of 37 pL/cell/day. For run 2, the CSPR never dropped below 50 pL/cell/day, with a minimum of 55 pL/cell/day. Online viable biomass measurement coupled with the bioreactor feeding rate can avoid such fluctuations in CSPR.^23^, ^24^ Glucose concentrations were analyzed and remained higher than 55 mM (data not shown) and thus did not appear to be limited during the run. However, the limitation of other nutrients, such as cysteine,^14^ could not be ruled out. Screening for optimal CSPR values should be performed to get more insight into the impact of nutrient availability on cell culture performance. Cell retention within the reactor is crucial during perfusion to minimize the loss of cells through the harvest flow. The separation efficiency of the acoustic cell retention device was always higher than 90% and >94% during the first days of perfusion (Figure 4d) for both runs. However, after baculovirus infection, the liquid in the acoustic chamber was occasionally opaque, indicating poor separation. The separation efficiencies demonstrated a decrease towards the end of the culture (Figure 4d). This is likely caused by a change in the morphology of the insect cells after baculovirus infection and during cell death. Since acoustic cell separation is based on particle size and physical properties of the cells, changing cell morphology can affect cell separation.^25^

**TABLE 1:** Green fluorescent protein productivity comparison between two perfusion runs with baculovirus infection versus a batch run.

	MOI	Run time (days)	Maximum viable cell concentration (cells/mL)	Product yield per cell (μg/cell)	Volumetric productivity (mg/L/day)	Yield of product per medium (μg/mL)
Run 1	0.01	12.2	2.77 × 10^7^	1.01 × 10^−5^	23 ± 1.9	28.0 ± 2.3
Run 2	0.01	16.8	2.46 × 10^7^	1.01 × 10^−5^	14.8 ± 1.2	15.7 ± 1.3
Run 2 shortened	0.01	11.9	2.46 × 10^7^	9.87 × 10^−6^	20.5 ± 1.7	22.5 ± 1.9
Batch reference	1	3.8	4.0 × 10^6^	4.23 × 10^−6^	4.5 ± 0.4	16.9 ± 1.4

*Note*: For run 2, two termination time points were included to indicate the effect on volumetric productivity and medium use and for comparison to run 1. ± Values indicate the standard measurement errors.

#### 3.4.2 Development of baculovirus infection

eGFP fluorescence was used to determine the percentage of cells infected with Ac‐2FL. Cell growth stopped around 2 days post‐infection (dpi), indicating all cells were infected. This is in accordance with the moment GFP expression becomes visible about 3 days post‐infection or 1 day after the majority of the cells became infected (Figure 4b). Infection slowed down after the percentage of infected cells reached 60%–70% around day 8 (4 dpi). This was unexpected, as in batch cultures a continuous rapid increase towards almost 100% infected cells is achieved.

This could be due to the presence of defective particles causing co‐infections and a decrease in GFP expression. The TCID50 used here was done using the Expi‐Sf cells and scoring on the GFP expression of the virus and thus only detects virus particles containing the GFP gene. Based on these final titers reached and the maximum infected cell concentration a value of 2 budded viruses released per cell is calculated., where normally values between 50 and 400 BV per cell are obtained. This supports the hypothesis of defective particles being present that interfere with GFP production. Moreover, the final titers for baculovirus containing the GFP gene of 3.5–5 × 10^7^ TCID_50_/mL result in an MOI between 1 and 2, which would result in about 60%–80% infection with GFP containing virus particles, which agrees with the data. The slow infection could also be related to the high cell concentration, which is known to reduce the virus binding efficiency, potentially due to cell–cell interactions resulting in clumping.^26^ Some cell clumping was observed in both reactor runs. Increasing the agitation speed could reduce clumping, although higher agitation rates can negatively impact cell growth.^27^ Furthermore, baculovirus expression is influenced by the cell cycle phase upon infection.^28^ It has been demonstrated that infection during the G_1_ and S phases results in faster and higher protein expression and that cells arrested in the G2 phase of the cell cycle are less susceptible to baculovirus infection.^29^ Specific growth rates were lower in both cultures after day 3 just before infection. This may have resulted in the accumulation of cells in the G2 phase and thus a slower infection progression in part of the cells. However, measurement of cell cycle distribution is difficult in infected cultures due to interference of baculovirus DNA. Finally, the viability started to drop rapidly around day 8. Dead cells were excluded from the analysis and consequently, the percentage of cells not yet showing green fluorescence will increase due to the loss of infected cells from the measurement which could explain the slow increase in the percentage of GFP‐containing cells after this point. This lower viability could also have resulted in virus degradation. Figure 4c shows the specific productivity. After day 11 the calculation of the specific productivity became very inaccurate due to the low viable cell concentrations. Until day 11 the specific productivity was constant over time and comparable for both runs.

The virus titers showed a similar profile for both runs (Figure 4c). Starting at 2 days post‐infection, titers increased until maximum infectious titers of 3.5–5.0 × 10^7^ TCID_50_/mL were reached at day 8. Furthermore, it can be seen that the increase is slightly later for run 2 as compared to run 1, which agrees with the later decrease in viability observed earlier. This could be caused by the slightly higher CCI in the first run or slight differences in MOI, which further demonstrates the relationship between CCI and MOI for the timing of the run.

#### 3.4.3 Volumetric productivity

After infection with Ac‐GFP, total GFP concentrations increased from 3 to 4 days post‐infection and this is in line with the detection of GFP inside the cells around day 3 post‐infection. Maximum concentrations of 230–280 mg/L were reached at 6 days post‐infection (Figure 4c). The GFP concentration increased no further 6 days post‐infection due to the low concentration of viable cells present after that day. The GFP yield per cell was very comparable for both perfusion runs (Table 1). Comparison of the perfusion runs infected at high cell concentration with batch reference runs infected with the same baculovirus construct showed that yield per cell was improved by 2‐fold for the perfusion cultures and that the volumetric productivity was 3–5 fold higher (Table 1). The average product yield per medium was dependent on the run time. A shorter run time improved the volumetric productivity and the product yield per medium (Table 1). Decreasing or stopping the perfusion flow towards the end of the culture can further improve product yield per medium and reduce medium consumption. Caron et al.^18^ did perfusion cultures with Sf‐9 cells and a perfusion rate of 1–1.5 reactor volumes per day. Cultures were infected around 1.2 × 10^7^ cells/mL with an MOI of 2–4 using a baculovirus containing the gene of the VP6 protein of bovine rotavirus. A slight drop of 25% in cell specific yield was observed as compared to batch. However, due to the higher CCI the volumetric productivity increased with a factor 2. Chico et al.^15^ ran perfusion cultures with Tn5 cells infected at different CCIs (2–4 × 10^6^ cells/mL). Perfusion rates depended on the cell concentration and were between 1 and 3 reactor volumes per day. A recombinant Autographa californica nucleopolyhedrovirus expressing the human β‐trace glyco‐protein was used. Specific productivity in the perfusion cultures was comparable to the maximal value found in batch at a value of 86 μg/10^6^ cells/day. Volumetric productivity increased proportional with the increase in infected cell concentration from 54 mg/L/day to 1.2 g/L/day. Zhang et al.^30^ used an acoustic separation device to run perfusion cultures of Sf‐9 cells. With perfusion rate of 1 reactor volume per day 3 10^7^ cells/mL were reached. Two baculoviruses, one containing the gene for human chitinase and one containing the gene for monocyte‐colony inhibiting factor were used for infection. However, cells were not infected in the bioreactor. Instead cells were taken from the perfusion cultures at the highest concentration and resuspended in fresh medium in spinner flasks and infected at a CCI of 2 × 10^6^ cells/mL and an MOI between 0.1 and 1 pfu/cell. The same was done with cells taken from the exponential phase of a batch culture. The protein production was comparable between the cells obtained from the perfusion and the batch cultivation. In conclusion, this data demonstrates the potential for rapid process intensification of the baculovirus expression vector system by using perfusion to infect insect cells at high cell concentrations and low MOI.

## 4 CONCLUSION

Process intensification was achieved for the baculovirus expression vector system by implementing perfusion to infect insect cells in stirred‐tank bioreactors at high CCI and low MOI. The investigated strategy used a low MOI of 0.01 to infect insect cells with baculovirus at viable cell concentrations of 1.0–1.2 × 10^7^ cells/mL. By using an acoustic perfusion device to allow for medium exchange and cell retention, the cell density effect was overcome and concentrations of 2.5–2.8 × 10^7^ viable cells/mL were achieved in two perfusion bioreactor runs that showed high reproducibility. The recombinant protein yield per cell did not decrease at these high cell concentrations and bioreactor volumetric productivity was up to 4.8‐fold higher compared to batch processes. Process optimization to reduce total cultivation time or reach even higher cell concentrations may further increase volumetric productivity. A low MOI strategy allows for the use of small virus stocks, which can be generated more efficiently and quickly than larger stocks. For example, using an MOI of 0.01 instead of 5 would allow for a 500 times reduction in viral inoculation volume. This can save time and resources, and reduce the risk of contamination. Implementing this perfusion strategy for the BEVS reduces the time for scale‐up and could increase the production capacity of existing production facilities.

## AUTHOR CONTRIBUTIONS

**Jort J. Altenburg:** Conceptualization (equal); data curation (equal); formal analysis (equal); investigation (equal); methodology (equal); project administration (equal); resources (equal); supervision (equal); validation (equal); visualization (lead); writing – original draft (lead). **Brenda E. Juarez‐Garza:** Investigation (equal); writing – review and editing (equal). **Jelle van Keimpema:** Investigation (equal); writing – review and editing (equal). **Linda van Oosten:** Investigation (equal); resources (equal); writing – review and editing (equal). **Gorben P. Pijlman:** Funding acquisition (equal); supervision (equal); writing – review and editing (equal). **Monique M. van Oers:** Funding acquisition (lead); resources (equal); supervision (equal); writing – review and editing (equal). **René H. Wijffels:** Funding acquisition (equal); project administration (equal); resources (equal); supervision (equal); writing – review and editing (equal). **Dirk E. Martens:** Conceptualization (equal); funding acquisition (equal); project administration (equal); resources (equal); supervision (equal); writing – review and editing (equal).

## CONFLICT OF INTEREST STATEMENT

The authors declare no conflict of interest.

## Data Availability

The data that support the findings of this study are available from the corresponding author upon reasonable request.

## References

[1] Shanmugaraj B , Phoolcharoen W . Addressing demand for recombinant biopharmaceuticals in the COVID‐19 era. Asian Pac J Trop Med. 2021;14(2):49‐51. doi:10.4103/1995-7645.306736

[2] Wolfe ND , Dunavan CP , Diamond J . Origins of major human infectious diseases. Nature. 2007;447(7142):279‐283. doi:10.1038/nature05775 17507975 PMC7095142

[3] Domínguez‐Andrés J , van Crevel R , Divangahi M , Netea MG . Designing the next generation of vaccines: relevance for future pandemics. MBio. 2020;11(6):1‐16. doi:10.1128/mBio.02616-20 PMC8534290 33443120

[4] Possee RD . Baculoviruses as gene expression vectors. Ann Rev Microbiol. 1988;42:177‐199. doi:10.1146/annurev.mi.42.100188.001141 3059993

[5] Carinhas N , Bernal V , Oliveira R , Alves PM . Baculovirus production for gene therapy : the role of cell density, multiplicity of infection and medium exchange. Appl Microbiol Biotechnol. 2009;81(6):1041‐1049. doi:10.1007/s00253-008-1727-4 18923829

[6] Maranga L , Brazao TF , Carrondo MJT . Virus‐like particle production at low multiplicities of infection with the baculovirus insect cell system. Biotechnol Bioeng. 2003;84(2):245‐253. doi:10.1002/bit.10773 12966582

[7] Sequeira DP , Correia R , Carrondo MJT , Roldão A , Teixeira AP , Alves PM . Combining stable insect cell lines with baculovirus‐mediated expression for multi‐HA influenza VLP production. Vaccine. 2018;36(22):3112‐3123. doi:10.1016/j.vaccine.2017.02.043 28291648

[8] Gallo‐Ramírez LE , Nikolay A , Genzel Y , Reichl U . Bioreactor concepts for cell culture‐based viral vaccine production. Expert Rev Vaccines. 2015;14(9):1181‐1195. doi:10.1586/14760584.2015.1067144 26178380

[9] Jäger V . Perfusion bioreactors for the production of recombinant proteins in insect cells. Current Applications of Cell Culture Engineering. Dordrecht: Springer; 1996:191‐198. doi:10.1007/0-306-46850-6_16 22358483

[10] Bernal V , Carinhas N , Yokomizo AY , Carrondo MJT , Alves PM . Cell density effect in the baculovirus‐insect cells system: a quantitative analysis of energetic metabolism. Biotechnol Bioeng. 2009;104(1):162‐180. doi:10.1002/bit.22364 19459142

[11] Pijlman GP , Grose C , Hick TAH , et al. Relocation of the atttn7 transgene insertion site in bacmid dna enhances baculovirus genome stability and recombinant protein expression in insect cells. Viruses. 2020;12(12):1‐17. doi:10.3390/v12121448 PMC7765880 33339324

[12] Ikonomou L , Schneider YJ , Agathos SN . Insect cell culture for industrial production of recombinant proteins. Appl Microbiol Biotechnol. 2003;62(1):1‐20. doi:10.1007/s00253-003-1223-9 12733003

[13] Lavado‐García J , Pérez‐Rubio P , Cervera L , Gòdia F . The cell density effect in animal cell‐based bioprocessing: questions, insights and perspectives. Biotechnol Adv. 2022;60:1‐14. doi:10.1016/j.biotechadv.2022.108017 35809763

[14] Radford KM , Reid S , Greenfield PF . Substrate limitation in the baculovirus expression vector system. Biotechnol Bioeng. 1997;56(1):32‐44. doi:10.1002/(SICI)1097-0290(19971005)56:1<32::AID-BIT4>3.0.CO;2-W 18636607

[15] Chico E , Jäger V . Perfusion culture of baculovirus‐infected BTI‐Tn‐5B1‐4 insect cells: a method to restore cell‐specific β‐trace glycoprotein productivity at high cell density. Biotechnol Bioeng. 2000;70(5):574‐586. doi:10.1002/1097-0290(20001205)70:5<574::AID-BIT12>3.0.CO;2-Q 11042554

[16] Caron AW , Tom RL , Kamen AA , Massie B . Baculovirus expression system scaleup by perfusion of high‐density Sf‐9 cell cultures. Biotechnol Bioeng. 1994;43(9):881‐891. doi:10.1002/BIT.260430907 18615881

[17] Massotte D . G protein‐coupled receptor overexpression with the baculovirus‐insect cell system: a tool for structural and functional studies. Biochim Biophys Acta Biomembr. 2003;1610(1):77‐89. doi:10.1016/S0005-2736(02)00720-4 12586382

[18] Caron AW , Archambault J , Massie B . High‐level recombinant protein production in bioreactors using the baculovirus–insect cell expression system. Biotechnol Bioeng. 1990;36(11):1133‐1140. doi:10.1002/bit.260361108 18595054

[19] Yovcheva M , Thompson K , Barnes S , et al. High‐titer recombinant protein production. Genet Eng Biotechnol News. 2018;38(13):20‐21. doi:10.1089/gen.38.13.08

[20] Schulze M , Lemke J , Pollard D , Wijffels RH , Matuszczyk J , Martens DE . Automation of high CHO cell density seed intensification via online control of the cell specific perfusion rate and its impact on the N‐stage inoculum quality. J Biotechnol. 2021;335(February):65‐75. doi:10.1016/j.jbiotec.2021.06.011 34090946

[21] Dalm MCF , Cuijten SMR , Van Grunsven WMJ , Tramper J , Martens DE . Effect of feed and bleed rate on hybridoma cells in an acoustic perfusion bioreactor: part I. Cell density, viability, and cell‐cycle distribution. Biotechnol Bioeng. 2004;88(5):547‐557. doi:10.1002/bit.20287 15459904

[22] Fernandes B , Correia R , Alves PM , Roldão A . Intensifying continuous production of gag‐HA VLPs at high cell density using stable insect cells adapted to low culture temperature. Front Bioeng Biotechnol. 2022;10:977. doi:10.3389/FBIOE.2022.917746/BIBTEX PMC9277389 35845394

[23] Carvell JP , Dowd JE . On‐line measurements and control of viable cell density in cell culture manufacturing processes using radio‐frequency impedance. Cytotechnology. 2006;50(1–3):35‐48. doi:10.1007/s10616-005-3974-x 19003069 PMC3475999

[24] Dowd JE , Jubb A , Kwok KE , Piret JM . Optimization and control of perfusion cultures using a viable cell probe and cell specific perfusion rates. Cytotechnology. 2003;42(1):35‐45. doi:10.1023/A:1026192228471 19002926 PMC3449505

[25] Ding X , Peng Z , Lin SCS , et al. Cell separation using tilted‐angle standing surface acoustic waves. Proc Natl Acad Sci USA. 2014;111(36):12992‐12997. doi:10.1073/pnas.1413325111 25157150 PMC4246961

[26] Power JF , Reid S , Greenfield PF , Nielsen LK . The kinetics of baculovirus adsorption to insect cells in suspension culture. Cytotechnology. 1996;21(2):155‐163. doi:10.1007/BF02215665 22358665

[27] Kioukia N , Nienow AW , Al‐Rubeai M , Emery AN . Influence of agitation and sparging on the growth rate and infection of insect cells in bioreactors and a comparison with hybridoma culture. Biotechnol Prog. 1996;12(6):779‐785. doi:10.1021/bp9600703

[28] Saito T , Dojima T , Toriyama M , Park EY . The effect of cell cycle on GFPuv gene expression in the baculovirus expression system. J Biotechnol. 2002;93(2):121‐129. doi:10.1016/S0168-1656(01)00398-4 11738719

[29] Lynn DE , Hink WF . Infection of synchronized TN‐368 cell cultures with alfalfa looper nuclear polyhedrosis virus. J Invertebr Pathol. 1978;32(1):1‐5. doi:10.1016/0022-2011(78)90167-2

[30] Zhang J , Collins A , Chen M , Knyazev I , Gentz R . High‐density perfusion culture of insect cells with a biosep ultrasonic filter. Biotechnol Bioeng. 1998;59(3):351‐359. doi:10.1002/(SICI)1097-0290(19980805)59:3<351::AID-BIT11>3.0.CO;2-H 10099346

